# Clinical analysis of kasabach-merritt syndrome in 17 neonates

**DOI:** 10.1186/1471-2431-14-146

**Published:** 2014-06-11

**Authors:** Ping Wang, Wei Zhou, Li Tao, Ning Zhao, Xiao-Wen Chen

**Affiliations:** 1 Department of Neonatology, Guangzhou Women and Children's Medical Center, Jinan University, Renminzhong Road 318, Guangzhou 510120, China

**Keywords:** Arterial embolization, Steroid therapy, Kasabach-Merritt syndrome, Neonate, Vincristine

## Abstract

**Background:**

Kasabach-Merritt syndrome (KMS) is characterized by giant hemangiomas and severe thrombocytopenia, which may result in life-threatening multi-organ hemorrhage. This study evaluated the clinical characteristics, treatments, and outcomes in neonates with KMS, in order to find out the optimal therapy.

**Methods:**

The clinical data of 17 patients treated for KMS in the Department of Neonates, Guangzhou Women and Children’s Medical Center, Guangzhou Medical University, China from January 2007 to January 2012 were retrospectively analyzed.

**Results:**

The patients were 13 males and 4 females, aged 17 hours to 28 days at admission. Four patients had visceral hemangiomas and 13 had cutaneous hemangiomas. All had thrombocytopenia and coagulation disorders. Intravenous steroid therapy was initially effective in 6 patients (of which 3 relapsed) and ineffective in 11. The 11 patients with a poor response to steroids and the 3 who relapsed underwent arterial embolization therapy, which was effective in 9 patients (of which 1 relapsed), ineffective in 4, and discontinued before completion in 1. Subsequently, four patients in whom arterial embolization therapy was ineffective and one with relapse were treated with vincristine. This was effective in four patients, and the other died of disseminated intravascular coagulation. Steroid therapy was effective in 35.3% of patients, but the relapse rate was 50%. Arterial embolization was effective in 64.3% of patients and vincristine was effective in 80%.

**Conclusions:**

In patients with neonatal KMS, steroid therapy has a low rate of effectiveness and high rate of relapse. Arterial embolization has a good rate of effectiveness. Combined steroid and embolization therapy should be considered for first-line treatment of neonatal KMS. If this approach is ineffective, vincristine may be useful.

## Background

Kasabach-Merritt syndrome (KMS), also known as giant hemangioma with thrombocytopenia, was first reported by Kasabach and Merritt in 1940. KMS accounts for about 1% of cases of hemangioma. KMS is characterized by giant hemangiomas and severe thrombocytopenia, which may result in life-threatening multi-organ hemorrhage. About 80% of patients present within 1 year after birth, and the reported mortality rate ranges from 10% to 37% [1]. However, the optimal therapy for neonatal KMS is currently unclear. From January 2007 to January 2012, 17 patients with KMS were admitted to the Department of Neonates at Guangzhou Women and Children’s Medical Center in China.

## Methods

### Subjects

From January 2007 to January 2012, 17 patients with giant hemangioma who were eventually diagnosed with KMS were admitted to the Department of Neonates at Guangzhou Women and Children’s Medical Center, China.

### Diagnostic criteria

The diagnostic criteria for KMS were as follows: (1) hemangioma of the skin or internal organs; (2) thrombocytopenia and consumptive coagulopathy; (3) hemangioma confirmed by B-mode ultrasonography, color Doppler flow imaging, computed tomography (CT), or magnetic resonance imaging (MRI); (4) other causes for the abnormalities excluded, such as hypersplenism or idiopathic thrombocytopenic purpura.

The diagnostic criteria for disseminated intravascular coagulation (DIC) followed overt DIC criteria by ISTH (Table 1).

**Table 1:** The diagnostic criteria of DIC by ISTH

	**Overt DIC by ISTH**	
Platelet Count	50,000 ~ 100,000/ul	1 point
	<50,000/ul	2 points
PT	Prolongation of PT	
	3 ~ 6 sec	1 point
	> 6 sec	2 points
Fibrinogen	< 100 mg/dl	1 point
D-dimer	0.5 – 1	1 point
	1 – 2	2 points
	> = 2(μg/ml)	3 points
Total	Overt DIC > =5 points	

### Consent

Written informed consents were obtained from the patients for publication of this case report and any accompanying image. Copies of the written consents are available for review by the Editor of this journal.

### Ethics

The research has been approved by Guangzhou Women and Children’s Medical Center’s appropriate ethics committee.

### Clinical features

The appearances, characteristics, and complications of hemangioma in the 17 neonates with KMS were summarized and analyzed.

### Therapeutic methods

The 17 neonates with KMS were treated with steroid therapy, arterial embolization, and vincristine, depending on their individual responses to treatment.

## Results

### Patient characteristics

The 17 patients with KMS included 13 males and 4 females. Their age at admission ranged from 17 hours to 28 days. One patient was born prematurely (34^+5^ weeks gestation), and the remainder were born at term. The birth weight ranged from 2300 g to 4000 g.

### Clinical manifestations

**Figure 1:**
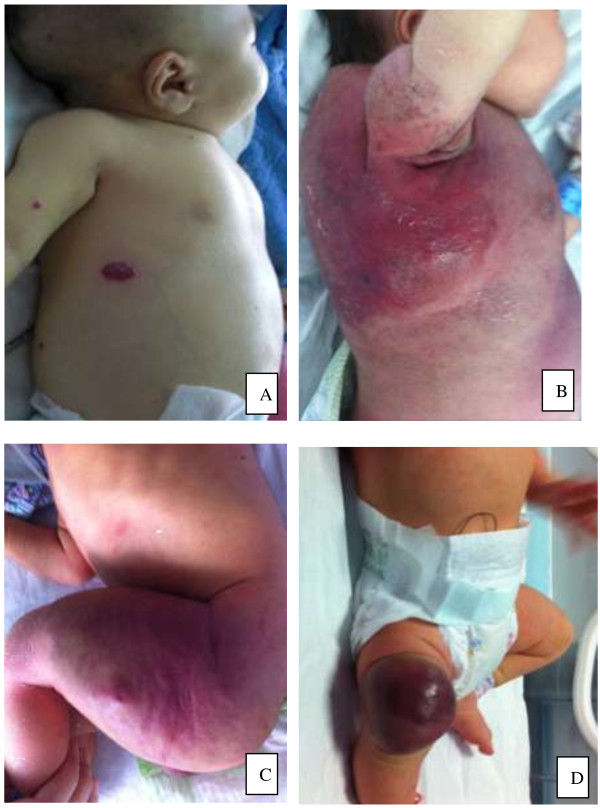
**Images of four patients. (A)** The patient with multiple cutaneous hemangiomas (more than 20 hemangiomas of different sizes) and multiple hepatic hemangiomas. **(B)** The patient with cutaneous hemangiomas on back and axilla. **(C)** The patient with cutaneous hemangiomas on left leg. (**D)** The patient with cutaneous hemangiomas on right knee.

Of the 17 patients, 4 had visceral hemangiomas, including 1 patient with multiple cutaneous hemangiomas (more than 20 hemangiomas of different sizes) and multiple hepatic hemangiomas, 1 patient with a right submandibular racemose hemangioma, 1 patient with a giant hepatic hemangioma, and 1 patient with an intracranial hemangioma. The remaining 13 patients had cutaneous hemangiomas only. The locations of cutaneous hemangiomas varied widely. Cutaneous hemangiomas appeared as violet or dark red lesions with obvious swelling, increased tension, slightly increased skin temperature, unclear boundaries, and a firm or hard texture (Figure 1). Seven patients had large numbers of bleeding points and ecchymosis. In one case, right submaxillary ecchymosis and thrombocytopenia were detected during the neonatal period, leading to a diagnosis of thrombocytopenic purpura at the local hospital. The patient was transferred to Guangzhou Women and Children’s Medical Center for treatment of a pericardial effusion. CT showed a racemose hemangioma of the pericardium, and B-mode ultrasonography showed a right submaxillary subcutaneous hemangioma. In another case, the patient was jaundiced at admission, and the right eye protruded further than the left eye. The patient had a black rash on his right upper eyelid, and a large amount of ascites. Brain MRI showed a hemangioma arising from a vascular malformation in the right cavernous sinus and right eye socket.

### Laboratory examination findings

Laboratory examination findings showed a mean platelet count of 31.1 ± 21.7 × 10^9^/L (range 2–119 × 10^9^/L), hemoglobin concentration of 94.1 ± 21.1 g/L (range 66–128 g/L), prothrombin time of 28 ± 5.6 s (range 13–38 s) with a prolonged prothrombin time in 15 patients, thrombin time of 18 ± 3.6 s (range 15–28 s) with a prolonged thrombin time in 5 patients, activated partial thromboplastin time of 51 ± 5.8 s (range 40–72 s) with a prolonged activated partial thromboplastin time in 8 patients, and fibrinogen level of 0.8 ± 0.37 mg/dL (range 0.34-1.8 mg/dL) with a low fibrinogen level in 15 patients. Two patients met the diagnostic criteria for DIC.

### Treatment

The 17 patients received sequential combined therapy. The initial therapy was supportive. Six patients with a platelet count of <20 × 10^9^/L received apheresis platelet infusions. Eight patients with a fibrinogen level of <1.0 mg/dL received cryoprecipitate infusions. Intravenous dexamethasone 1 mg/kg/day was administered simultaneously as first-line drug treatment. The platelet count started to increase at 3–8 days after the start of treatment in seven patients, reaching a normal value at 16–28 days. Coagulation function improved, and no bursting or bleeding of hemangiomas was observed. After the platelet count returned to normal, we evaluated the effectiveness of the therapeutic regimen. If the treatment was effective, it was gradually withdrawn over the following 1–2 months, after which patients were discharged on medication. The six patients who responded well to steroid therapy were regularly followed up at the hospital. In three patients, the platelet counts remained normal for the following year. The hemangiomas did not enlarge or bleed, and regressed within 1–2 years. In the other three patients, the platelet counts subsequently decreased (at 30 days, 3 months, and 5 months after treatment), and they were re-admitted to hospital and underwent arterial embolization. No relapses were observed over the following year.

**Figure 2:**
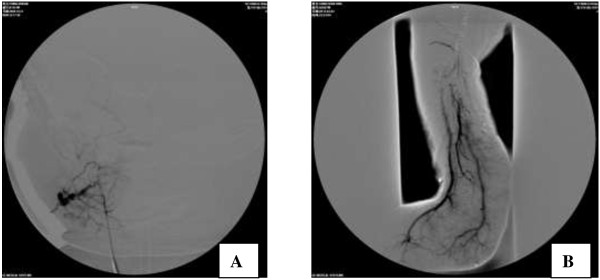
**Images of arterial embolization. (A)** Right submaxillary hemangioma. **(B)** Right leg and ankle hemangioma.

**Figure 3:**
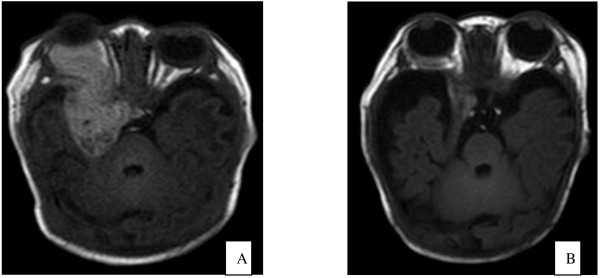
**MRI of the intracranial hemangioma in one case with a huge intracranial hemangioma before and after treatment. (A)** Exophthalmos of the right eye and a huge intracranial hemangioma. **(B)** Reduction of exophthalmos of the right eye, and regression of the intracranial hemangioma.

The other 11 patients received supportive treatment and steroid therapy for 10 days, but their platelet counts remained low, and the hemangiomas bled in some cases. One patient discontinued therapy because of the severity of disease and financial difficulty. The remaining 10 patients underwent arterial embolization. The femoral artery was cannulated under general anesthesia using the Seldinger technique. A 3-F or 4-F cannula was introduced and the patient was heparinized. A 3-F or 4-F Cobra catheter was introduced for arteriography, and was advanced to the feeding artery of the hemangioma. Arterial embolization was performed with a mixture of bleomycin A5 (8–12 mg/m^2^), iodinated oil (2 mL), and dexamethasone (2 mg). After removal of the catheter, the bleeding was controlled by direct pressure for 15 min, and a pressure dressing was applied (Figure 2). Six patients were given dexamethasone after arterial embolization. The platelet counts increased at 2–5 days after embolization, and reached normal levels at 10–16 days. The coagulation function was improved, and no bursting or bleeding of hemangiomas was observed. Patients were followed up regularly after embolization. Only one patient developed recurrence at 5 months after embolization, and underwent repeat embolization and vincristine chemotherapy (1 mg/m^2^ weekly). In the remaining four patients, the platelet count decreased at 1 week after surgery. These patients were treated with vincristine chemotherapy 1 mg/m^2^ weekly, resulting in improvement. Three of these patients did not relapse during a year of follow-up, and the other patient died of severe disseminated intravascular coagulation. In one patient with intracranial hemangioma, the hemangioma regressed during 2 years of follow-up (Figure 3). One patient developed a fever during treatment, and one developed gastrointestinal adverse effects. No cases of bone marrow depression or neurotoxicity were observed.

## Discussion

The main diagnostic features of KMS are giant hemangioma and decreased platelet count. Giant cutaneous hemangiomas are easy to diagnose on physical examination. Visceral hemangiomas are easily missed, and patients may present with large ecchymoses. According to the cases in this research, it illustrates that KMS should be considered in children with unexplained thrombocytopenia and coagulation disorders. Routine blood testing showed varying degrees of low hemoglobin concentration, low plasma fibrinogen level, and prolonged prothrombin time. B-mode ultrasonography, CT, and MRI showed the size, appearance, and layers of hemangiomas, as well as their relationships with peripheral vessels, and distinguished hemangiomas from vascular malformations.

KMS is associated with Kaposi haemangioendothelioma(KHE) in over 90% of cases according to the research findings recently, and uncommonly with infantile and congenital haemangioma. The MRI findings of KHE have some characters. It presents as an enhancing, ill-defined, soft-tissue mass that is hypointense or isointense on T1 weighted images and hyperintense on T2 weighted images compared with muscle. Prominent vascular channels, presenting as flow voids in the tumour or as linear enhancing channels adjacent to the tumour, are usually noted [2]. However, these findings are not specific for KHE. KHE can’t be confirmed by MRI only. The identification of hemangioma type rely mainly on pathologic examination, but KMS is associated with coagulation dysfunction, which is a contraindication of pathological biopsy. So our patients accepted B-mode ultrasonography, color Doppler flow imaging, or computed tomography (CT) because of the cheaper costs. Only one patient accepted MRI.

There are currently no consensus guidelines for the treatment of KMS. Some studies have reported good therapeutic effects with comprehensive sequential therapy, including steroid therapy, interferon, arterial embolization, vincristine, radiotherapy, and surgery [3-5]. There are no specific guidelines for diagnosis and treatment during the neonatal period.

In infants, glucocorticoid therapy is considered to be a good choice for initial drug treatment, because it can inhibit fibrinolysis and thrombosis, stimulate hematopoiesis in the bone marrow to increase release of platelets into the bloodstream, decrease the level of anti-platelet antibodies, and increase platelet count. However, it is only effective in 30% to 50% of patients with KMS [6,7]. Glucocorticoid therapy may be administered orally, intravenously, or locally; and commonly starts to show effectiveness at 1–2 weeks after the onset of treatment [8]. Our patients were treated with intravenous dexamethasone 1 mg/kg/day, and the platelet count started to increase after 3–8 days. In this study, steroid therapy was effective in 35.3% of patients, but the relapse rate was high (50%), resulting in an eventual effectiveness rate of only 17.6%. A more effective therapeutic approach is therefore necessary.

In patients who responded poorly to steroid therapy, arterial embolization was performed with bleomycin A5 (8–12 mg/m^2^), iodinated oil (2 mL), and dexamethasone (2 mg). Arterial embolization has been used to treat vascular tumors for many years [9,10], and is useful for controlling symptoms and promoting regression of hemangiomas. However, neonates have tiny vessels, and do not tolerate such intervention well. There have been a few previous reports of treatment by arterial embolization in neonates with KMS in China, especially in patients with coagulation disorders. The dose of contrast agent needed, risk associated with general anesthesia, and difficulty in catheter placement limit the usefulness of this treatment in neonates. In the present study, arterial embolization was effective in 64.3% of patients. A previous study of arterial embolization using bleomycin A5 found that partial embolization significantly improved coagulation function and decreased hemangioma blood flow, and relieved symptoms [11]. Another study found that local injection of urea destroyed the vascular endothelial cell matrix, suppressed endothelial cell growth, promoted endothelial cell atrophy, caused fibrosis of local tissue, hardened the tumor tissue, and caused thrombosis in the vascular lumen of the tumor body [12]. Local injection of urea has been used to successfully treat hemangiomas and vascular malformations, and may be useful for the treatment of neonatal KMS [12]. Arterial embolization in neonates is difficult and carries some risks, but can improve the patient’s condition if intravenous drug therapy has not controlled the hemangioma.

Intravenous vincristine has been used for the treatment of KMS, and can be used as a first-line drug in the treatment of Kaposiform hemangioendothelioma combined with KMS [13,14]. An American multicenter study found that vincristine therapy was useful in patients who were resistant to steroid and interferon therapy [13,15]. Vincristine, actinomycin, and cyclophosphamide can induce tumor regression and normalization of coagulation parameters [16]. Although vincristine is neurotoxic, it can help to alleviate symptoms in patients with KMS. In this study, vincristine was administered to five patients who responded poorly to steroid therapy and arterial embolization, and was effective in 80% of these patients. No adverse effects related to chemotherapy were observed. These results indicate that vincristine can be used to treat neonatal KMS. However, this study only had a small number of subjects, and a larger sample is needed to confirm the effectiveness of vincristine therapy. Haisley-Royster et al. [13] reported that platelet counts in patients with KMS increased to normal values after 5 weeks of vincristine therapy. Vincristine therapy cannot avoid hemangioma relapse, but has been reported to result in prolonged reduction in hemangioma growth [11], which is consistent with the findings of this study.

It is worth noting that platelet transfusion is suitable for increasing the platelet count urgently or preoperatively, but it cannot be used routinely, because the hemangioma will consume the platelets [5,17,18]. Moreover, platelet transfusion will promote an increase in hemangioma size by inducing blood coagulation [5,17,18]. Excessive platelet transfusion has been reported to aggravate KMS [5,17,18]. In this study, patients received apheresis platelet transfusions if their platelet count was <20 × 10^9^/L, which allowed us to perform arterial embolization. Transfusion therefore did not aggravate their condition.

Systemic interferon therapy has been used in patients who were resistant to steroid therapy, with good results [4,18]. However, the effectiveness of interferon therapy is variable. Interferon therapy can be considered as a second-line drug treatment that is effective in 50% to 60% of patients, but it only has weak inhibitory effects on endothelial cell and vascular growth, and continuous treatment is needed [19]. Continuous interferon therapy may result in increased transaminase levels, decreased platelet count, and decreased leukocyte count [20]. A meta-analysis by Michaud et al. [8] found that interferon therapy could cause spastic diplegia and dyskinesia in infants. They recommended that interferon therapy should not be used in patients aged less than 1 year, except when other treatment methods are ineffective and the patient’s condition is life-threatening [8].

Leong et al. [21] reported two neonates with KMS who received radiotherapy after full-dose steroid therapy and α-interferon therapy were ineffective, resulting in a rapid increase in platelet count. The hemangioma gradually regressed over 3 years in one patient, and fully regressed within 2 months in the other patient. No adverse effects associated with radiotherapy were detected during the follow-up periods of 8 and 5 years, respectively, suggesting that radiotherapy may be a safe and effective treatment for KMS. However, another study reported that radiotherapy for hemangioma may increase the frequency of secondary tumors [22]. The safety and effectiveness of radiotherapy for the treatment of KMS should be evaluated in further clinical studies with follow-up over a few decades.

## Conclusions

Neonatal KMS has a high relapse rate after steroid therapy. Arterial embolization has a good rate of effectiveness. Combined steroid therapy and arterial embolization can be used as first-line treatment for neonatal KMS. If this combination is ineffective, vincristine therapy may be useful.

## Abbreviations

KMS: Kasabach-Merritt syndrome; CT: Computed tomography; MRI: Magnetic resonance imaging; DIC: Disseminated intravascular coagulation; ISTH: International Society on Thrombosis and Haemostasis; KHE: Kaposi haemangioendothelioma.

## Competing interests

No benefits in any form have been received or will be received from any commercial party related directly or indirectly to the subject of this article.

## Author’s contributions

PW proposed the study and wrote the first draft. WZ guided the design of the study and helped to draft the manuscript. LT participated in its design and coordination and helped to revise the manuscript. NZ conceived of the study, and helped the acquisition and interpretation of data. XWC helped to draft the manuscript. All authors read and approved the final manuscript.

## Author’s information

Ping Wang is the first author of this paper.

## Pre-publication history

The pre-publication history for this paper can be accessed here:

http://www.biomedcentral.com/1471-2431/14/146/prepub
